# Influence of skill level on predicting the success of one's own basketball free throws

**DOI:** 10.1371/journal.pone.0214074

**Published:** 2019-03-22

**Authors:** Jonathan C. Maglott, David Chiasson, Peter B. Shull

**Affiliations:** State Key Laboratory of Mechanical System and Vibration, Shanghai Jiao Tong University, Shanghai, China; Texas A&M University, UNITED STATES

## Abstract

Basketball players sometimes claim to know when their shot is good, even before it goes in. This is likely because shooter proprioception can help determine shot outcome, even before their eyes confirm it. This phenomenon, however, has not been systematically explored for collegiate and recreational shooters. This study compared how well collegiate shooters and recreational shooters could predict outcomes of their own free throws without seeing the shot result. Forty collegiate and recreational shooters shot standard free throws while wearing liquid-crystal occlusion glasses that activated to occlude vision immediately following ball release during each shot. After each shot, shooters verbally predicted shot outcome as “in” or “out”, and predicted results were compared with actual outcomes. As anticipated, for made shots, collegiate shooters more accurately predicted their own shots than recreational shooters. However, unexpectedly, for missed shots, collegiate shooters were worse than recreational shooters and were even significantly worse than chance. Further analysis found that collegiate shooters exhibited a significantly higher bias toward predicting their shots as “in”. Understanding how shooters of different skill levels perceive their own shot could inform future training strategies for improving shooter accuracy.

## Introduction

Free throws are an important part of basketball as they can determine game outcome [1,2]. Previous research has explored optimal ball trajectory [2–5] and optimal shooter kinematics [6–8], as well as psychological factors related to basketball shooting [9–12] and theories for better coaching [1,13–15]. Deeper understanding of how people shoot can help coaches adopt more effective training methods.

Action anticipation, also known as success prediction, is the ability to predict the outcome of an event before receiving knowledge of results (KR) and is vital for decision making in sports [16]. In basketball, for example, anticipating that a shot will go in, bounce away from the basket, or miss the basket entirely typically affects the direction the player begins to move as the ball arcs through the air towards the rim. Action anticipation has been shown to be more pronounced in skilled athletes than novice athletes. For example, Aglioti et al. [17] showed that skilled shooters predict free throw outcomes with greater accuracy than novices when watching video clips of the free throws. In another study, Wu et al. [18] found that skilled basketball players exhibited better action anticipation than novice players because of superior visual perception techniques. While novices use "high-level decision-making strategies" to predict the outcome of an event, experts and skilled athletes rely on body cues [16,19].

Action anticipation accuracy is highest for one’s own actions. Cañal-Bruland et al. [20] found that the shooting players were better at judging their own shots as “in” compared to watching players. Similarly, Knoblich & Flach [21] showed that non-expert dart throwers judged the accuracy of their own throws better than the throws of others. When predicting the outcome of one’s own actions, athletes have an additional source of information known as proprioception. Proprioception is the awareness of one’s own body and spatial position independent of visual input. Previous work has shown that people are aware of their own movement errors because, during a movement, the brain compares actual proprioceptive feedback with a model of expected feedback and identifies differences as potential errors [22]. This awareness is evidenced by different cerebral areas activating when an error occurs in an action [23].

Proprioceptive feedback plays a necessary role in both learning new tasks [24] and adjusting existing skills [8]. Proprioception is particularly important for assessing complex movements like shooting a basketball free throw [25,26]. While Schmidt et al. [27] found that subjects performing a task tend to rely on KR rather than proprioceptive feedback, skilled athletes compared to novice athletes show less of a dependence on either KR or visual input for performance and performance assessment [28,29]. Müller and Abernathy [28] found that for cricket batsmen, the performance of skilled players was significantly less affected by vision occlusion when compared to low-skilled batsmen. In basketball, vision can even sometimes be subconsciously suppressed by skilled shooters [30]. While basketball shot prediction performance has been studied in a uniformly skilled group of players [20], it is unclear how varying skill level will influence performance in shot prediction across a spectrum of players.

This study sought to answer the question: how well can shooters of varying skill level predict the outcome of their own free throws? We hypothesized that: 1) collegiate shooters, as compared with recreational shooters, can more accurately predict *made free throws*, and 2) collegiate shooters, as compared with recreational shooters, can more accurately predict *missed free throws*. These are important, because understanding how shooters of different skill levels perceive their own shot could inform future training strategies for improving shooter accuracy.

## Methods

### Participants

Forty subjects participated in this study after giving informed written consent, including 20 collegiate basketball players (age: 20.8 ± 1.6 years, height: 1.83 ± 0.06 m, mass: 79.2 ± 9.2 kg) and 20 recreational basketball players (age: 23.2 ± 3.7 years, height: 1.75 ± 0.05 m, mass: 71.2 ± 12.4 kg). Collegiate players participated on a university basketball team and practiced under the direction of the university coaching staff. Recreational players participated in half-court pick-up basketball games recreationally one or more times each week. The Shanghai Jiao Tong University ethics committee approved this study, and all testing was conducted in accordance with the Declaration of Helsinki.

### Setup

Testing was conducted on a regulation basketball court with a standard 10 foot (3.05 m) high basketball goal from a standard free throw line 15 feet (4.57 m) from the backboard. Participants wore Plato liquid-crystal glasses (Translucent Technologies, Toronto, Canada) during the experiment. The LC glasses were controlled manually to occlude the participant’s vision after the ball was released [20]. Because the ball hitting the rim (or not hitting the rim) could give the shooter clues about the shot outcome, subjects also wore earphones that were connected to a smartphone which played constant white noise to occlude the shooter’s hearing during the experiment.

### Procedure

After warming up on their own, a baseline test was conducted where the participants shot 15 free throws and the number of made shots was recorded. After the baseline test, participants put on the glasses and earphones and shot 5 free throws with full vision to get familiar with the equipment. The participants then shot 3 sets of 10 free throws for a total of 30 free throws. Participants were instructed to make as many baskets as possible. Immediately after each shot while their vision was occluded, participants verbally indicated their prediction of the shot result: either “in” or “out”. Actual shot result and the participant’s prediction were recorded as “in” or “out” for post-testing analysis [17,20]. The participant received no feedback during the testing about the results or the accuracy of their predictions. Given that augmented KR can be a method for learning [27,31–33], all KR was withheld from the participant during the entire experiment. Shot results and predictions were manually recorded during each trial.

### Data analysis

True positive (correct prediction that a shot was in), false positive (incorrect prediction that a shot was in), true negative (correct prediction that a shot was out), and false negative (incorrect prediction that a shot was out) rates were calculated across all subjects. True positive and true negative rates were used to compare accuracy between groups. A two-sample t-test was performed to compare the correct (including both true positive and true negative) prediction rates of the two groups. Effect size was calculated as Cohen’s *d* [34].

Signal detection theory, commonly used in psychological research, was employed to further analyze the prediction accuracy of both groups. Signal detection theory is advantageous for discrimination studies, like the presented study, because it separates prediction accuracy from judgement bias [35,36]. Sensitivity, signal detection theory’s measure of accuracy, is the “ability of a participant to discriminate between two sets of stimuli (e.g. ‘ball was in’ or ‘ball failed to go in’)” [20]. Judgement bias is a participant’s likelihood to prefer one response over the other [20]. Sensitivity and judgement bias were calculated using the approach presented by Macmillan & Creelman [35] and used in previous studies [20,21,37]. After adjusting the raw shot counts up by 0.5 to eliminate any zeros (e.g. no made shots), true positive (TP) and false negative (FN) rates were calculated and converted to sensitivity and judgement bias scores via z-transformation: *sensitivity = z(TP)–z(FN)* and *bias = -0*.5 * (z(TP) + z(FN)) [35]. Two-sample t-tests were performed comparing the correct prediction rates, sensitivity, and bias between the two groups. For each t test, the degrees of freedom, t test value, and p values were recorded. Statistical significance was defined as p<0.05. Effect sizes were calculated as Cohen’s *d* [34].

## Results

**Fig 1:**
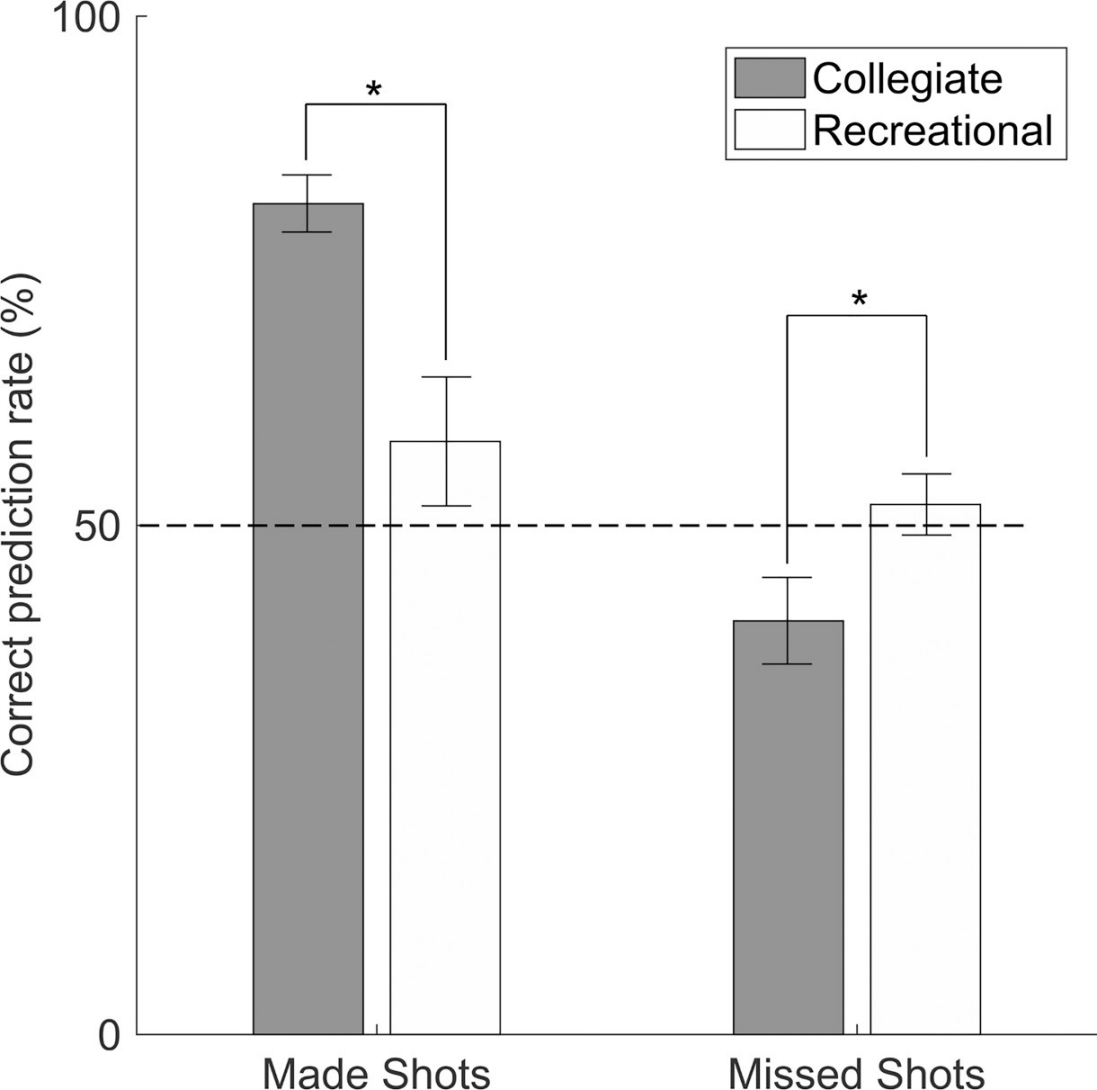
Correct prediction rates for the collegiate and recreational groups. (*) indicates statistically significant difference (p<0.05). Chance line shown at 50%. Bars indicate standard errors.

Collegiate shooters predicted missed shots worse than recreational shooters [t(38) = -2.2; *p* = .034; Cohen’s *d* = 0.70], and also worse than chance [t(38) = -2.2; *p* = .033; Cohen’s *d* = 0.70] (Fig 1). Collegiate shooters correctly predicted made shots better than recreational shooters by 24% (Table 1). Recreational shooters were not significantly better than chance at predicting made shots [t(38) = 1.3; *p* = .202; Cohen’s *d* = 0.41] or missed shots [t(38) = 0.68; *p* = .502; Cohen’s *d* = 0.21].

**Table 1:** Mean prediction rates by group.

	Collegiate		Recreational	
	Result In	Result Out	Result In	Result Out
Prediction In	82%^a^	59%^c^	58%^a^	48%^c^
Prediction Out	18%^b^	41%^d^	42%^b^	52%^d^
Total	100%	100%	100%	100%

^a^True positive

^b^False negative

^c^False positive

^d^True negative

**Fig 2:**
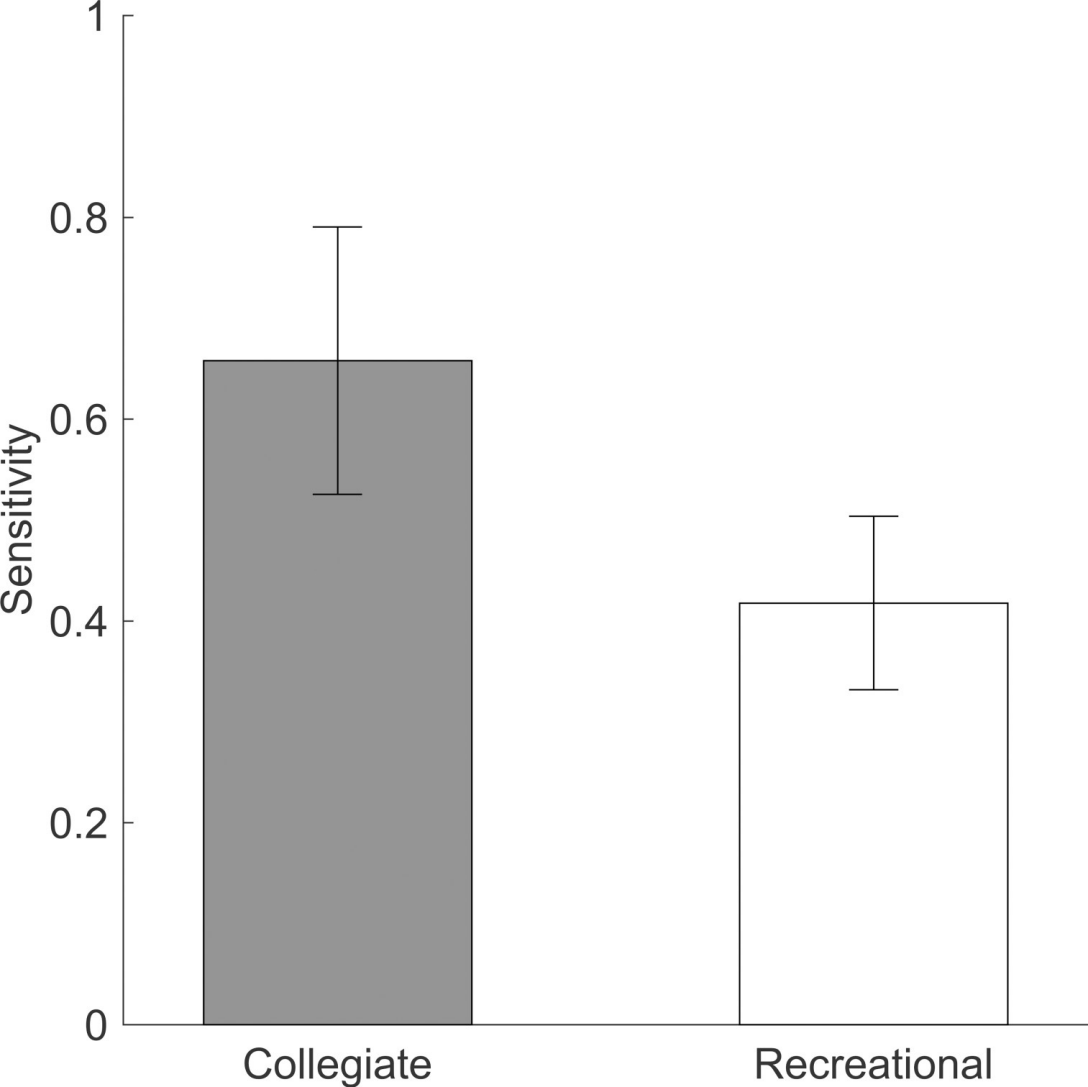
Sensitivity for both groups. Higher sensitivity indicates better ability to guess “in” or “out” with bias removed. Error bars indicate standard errors.

Collegiate shooters’ sensitivity in predicting the outcome of their own free throws was not significantly different than recreational shooters’ sensitivity [t(38) = -1.52; *p* = .137; Cohen’s *d* = 0.48] (Fig 2). The difference in mean sensitivity between collegiate and recreational shooters was 0.22.

**Fig 3:**
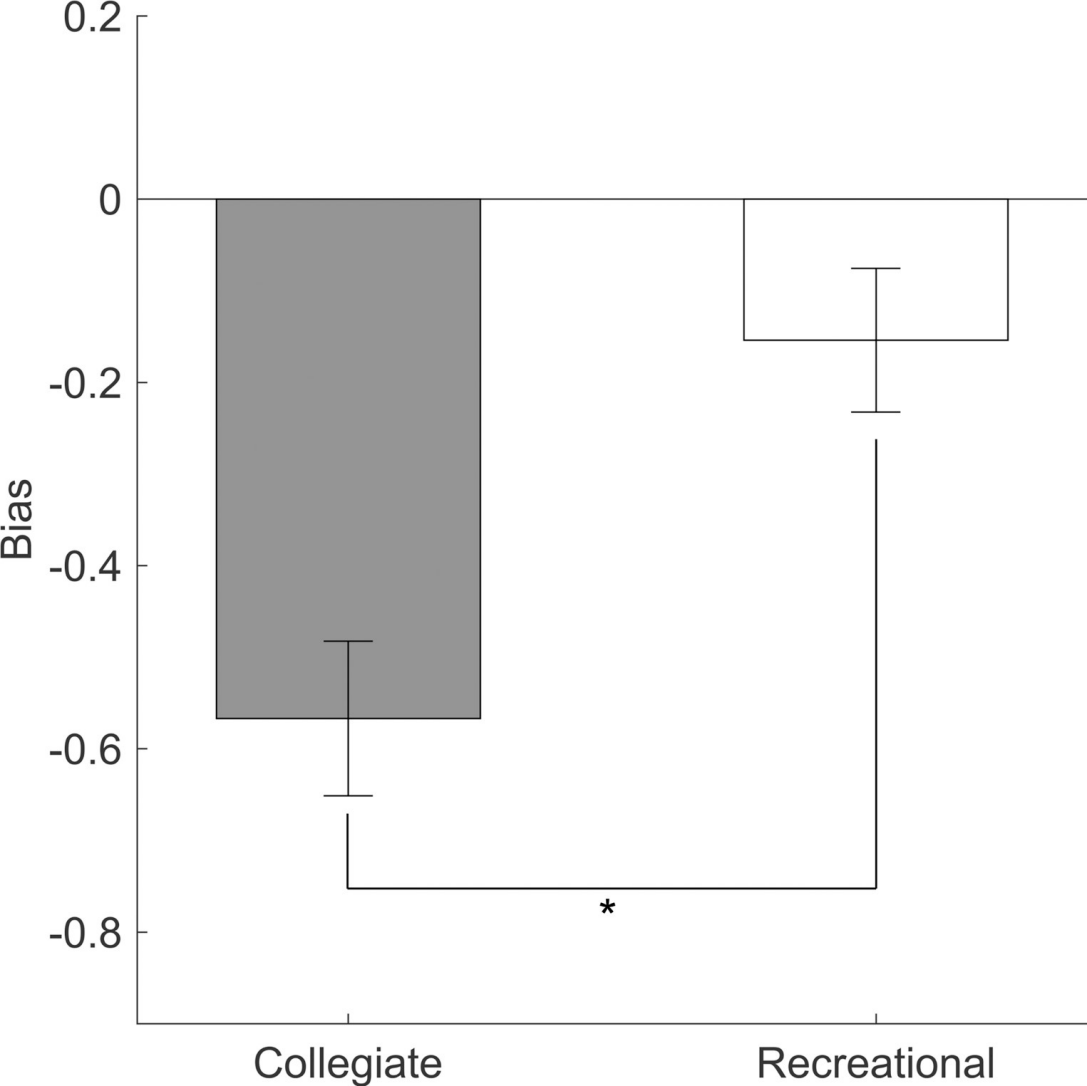
Judgmental bias for both groups. Negative judgement bias indicates a bias toward guessing “in” rather than “out”. (*) indicates statistically significant difference (p<0.05). Error bars indicate standard errors.

Collegiate shooters were significantly more biased toward predicting their own shots as “in” than the recreational shooters [t(38) = 3.58; *p* = .001; Cohen’s *d* = 1.13] (Fig 3). The mean difference in judgement bias between collegiate and recreational shooters was 0.41.

Collegiate shooters shot 26% better during the baseline test and 18% better during the experiment (Table 2). Baseline test performance was not a good predictor of experimental performance based on extremely large error (Table 2).

**Table 2:** Mean free throw accuracy.

Shooters	Baseline^a^	Experimental^a^	% Error^b^
Collegiate	65 ± 15%	50 ± 18%	26 ± 23%
Recreational	39 ± 16%	32 ± 17%	52 ± 69%

a. Total shots for baseline and experimental were 15 and 30, respectively.

b. % Error = absolute value ((Experimental–Baseline) / Baseline) * 100

## Discussion

The purpose of this paper was to determine the effect of skill level on a basketball shooter’s ability to predict the outcome of their own shot. In support of our first hypothesis, collegiate shooters were better than recreational shooters at predicting their own “in” free throw shots. However, results did not support our second hypothesis that collegiate shooters could predict the outcome of their own missed free throws more accurately than recreational shooters. Collegiate shooters not only predicted worse than recreational shooters, but they predicted worse than chance, meaning a simple coin toss at 50% would have been a better predictor of the collegiate shooters’ missed shots than the collegiate shooters themselves which were only correct 41% of the time (Table 1). Because the free throw should be a simple shot [5], collegiate shooters may expect to make each one, so they pay little attention to their proprioceptive feedback. The results of this study contradict findings by Rieser et al. [22] that suggest collegiate shooters should be able to identify errors in their shooting based on their body’s feedback. Collegiate shooters may have ignored or failed to detect errors in their proprioceptive feedback during missed shots because they typically rely on visual feedback for every shot and are not keenly aware of their own proprioceptive feedback. It is possible that collegiate shooters’ kinematic pattern was so consistent [38] for all shots that slight errors were undetected, and therefore all shots were perceived as good.

The discrepancy between collegiate shooters’ accuracy at predicting made and missed shots can be better explained using signal detection theory which provides distinction between sensitivity (the ability to discriminate between made and missed shots) and bias (the tendency to predict one outcome over the other)[35]. Signal detection theory analysis revealed that collegiate shooters had no greater sensitivity in predicting the outcome of their own free throws (Fig 2) compared to recreational shooters. However, they did exhibit much higher bias toward predicting their own shots as “in” when compared to the recreational shooter’s bias (Fig 3).

A similar sensitivity between the two groups does not support our original hypothesis that collegiate shooters would be better at predicting free throw outcomes. This similarity was surprising because during most recreational games a fouled player is awarded possession of the ball rather than a free throw, therefore we would expect recreational players to be less familiar with shooting (and predicting) free throws. These results also seem to contradict the suggestion by Aglioti et al. [17] that only experts have “the ability to discriminate between erroneous and correct performance.” This contradiction could be due to the collegiate participants in this study being less “elite” than the professional participants in the Aglioti et al. study. While collegiate players recruited for this study were not characterized as starters or reserve players because such a distinction was beyond the scope of this paper, it is likely that many reserve players would actually shoot free throws better than their starter teammates, as often starters are chosen for superior defensive abilities, athleticism, and height rather than free throw shooting ability.

The bias measured in this study was similar to the bias measured by Cañal-Bruland et al. [20] for skilled shooters shooting free throws. Collegiate shooters’ high bias toward predicting shots as “in” could be evidence of overconfidence in their own ability to make free throws. This overconfidence, or sometimes referred to as *desirability bias* [39], may be a useful tool used by the collegiate players to help them be more successful at shooting free throws [40]. However, overconfidence may impact the collegiate players during a game. For example, if they shoot a free throw and expect it to go in, but it doesn’t, they will not be as ready for a rebound as they might have been if they predicted the missed shot correctly. The expectation to make every free throw may induce accurate shooting but also unintentionally cause delayed attention to a rebound following a missed shot. The combination of collegiate players’ high bias and unremarkable sensitivity highlights a coach’s need to train free throw shooters to be ready for a rebound on every shot regardless of how they feel about the shot.

## Conclusions

This study found that collegiate shooters were more accurate than recreational shooters at predicting their own made shots, but worse than recreational shooters and chance at predicting their own missed shots. Collegiate shooters had similar sensitivity in predicting the outcome of their own shot when compared to recreational shooters, but exhibited much higher bias toward predicting their shots as “in”. Coaches could use vision-occlusion techniques, such as shooting free throws while blindfolded [13], to encourage better awareness of proprioceptive feedback in their players, because training shooters to be more aware of their own proprioception may increase their ability to predict shot outcomes and could potentially improve free throw performance [26].

## Supporting information

S1 Table Experimental data. (DOCX)

## References

[1] Kozar B, Vaughn R, Lord R, Whitfield K. Basketball free-throw performance: Practice implications. J Sport Behav. 1995;18: 123.

[2] Tran CM, Silverberg LM. Optimal release conditions for the free throw in men’s basketball. J Sports Sci. 2008;26: 1147–1155. 10.1080/02640410802004948 18645735

[3] Brancazio PJ. Physics of basketball. Am J Phys. 1981;49: 356.

[4] Okubo H, Hubbard M. Dynamics of the basketball shot with application to the free throw. J Sports Sci. 2006;24: 1303–1314. 10.1080/02640410500520401 17101533

[5] Hung GK, Johnson B, Coppa A. Aerodynamics and Biomechanics of the Free Throw. Biomedical Engineering Principles in Sports. Springer US; 2004 pp. 367–390.

[6] Miller S, Bartlett R. The relationship between basketball shooting kinematics, distance and playing position. J Sports Sci. 1996;14: 243–253. 10.1080/02640419608727708 8809716

[7] Okubo H, Hubbard M. Kinematics of arm joint motions in basketball shooting. Procedia Engineering. 2015 pp. 443–448.

[8] Khlifa R, Aouadi R, Hermassi S, Chelly MS, Jlid C, Gabbett TJ. Kinematic Adjustments in the Basketball Free Throw Performed with a Reduced Hoop Diameter Rim [Internet]. International Journal of Sports Science & Coaching. 2012 pp. 371–381. 10.1260/1747-9541.7.2.371

[9] Worthy D a, Markman AB, Maddox WT. Choking and Excelling at the Free Throw Line. Int J Creat Probl Solving. 2009;19: 53–58.

[10] Czech DR, Ploszay AJ, Burke KL. An Examination of the Maintenance of Preshot Routines in Basketball Free Throw Shooting. J Sport Behav. 2004;27: 323–329.

[11] Foster DJ, Weigand DA, Baines D. The Effect of Removing Superstitious Behavior and Introducing a Pre-Performance Routine on Basketball Free-Throw Performance. Journal of Applied Sport Psychology. 2006 pp. 167–171. 10.1080/10413200500471343

[12] Zestcott CA, Lifshin U, Helm P, Greenberg J. He Dies, He Scores: Evidence that Reminders of Death Motivate Improved Performance in Basketball. J Sport Exerc Psychol. 2016; 1–40. 10.1123/jsep.2016-0076 27736277

[13] Gilbert JN. Sport Psychology Teaching Approaches for High School Coaches and Their Student-Athletes. J Phys Educ Recreat Danc. 2017;88: 52–58. 10.1080/07303084.2016.1260076

[14] Okazaki VHA, Rodacki ALF, Satern MN. A review on the basketball jump shot. Sport Biomech. 2015;14: 190–205. 10.1080/14763141.2015.1052541 26102462

[15] Barney D, McGaha P. Where’s the B. E. E. F? Everyone Can Be A Shooter: Shooting Fundamentals and Activities Revisited. Strategies. 2006;19: 34–36.

[16] Abreu AM. Action anticipation in sports: A particular case of expert decision-making. Trends Sport Sci. 2014;21: 5–11.

[17] Aglioti SM, Cesari P, Romani M, Urgesi C. Action anticipation and motor resonance in elite basketball players. Nat Neurosci. 2008;11: 1109–1116. 10.1038/nn.2182 19160510

[18] Wu Y, Zeng Y, Zhang L, Wang S, Wang D, Tan X, et al The role of visual perception in action anticipation in basketball athletes. Neuroscience. 2013;237: 29–41. 10.1016/j.neuroscience.2013.01.048 23384606

[19] Abreu AM, Macaluso E, Azevedo RT, Cesari P, Urgesi C, Aglioti SM. Action anticipation beyond the action observation network: A functional magnetic resonance imaging study in expert basketball players. Eur J Neurosci. 2012;35: 1646–1654. 10.1111/j.1460-9568.2012.08104.x 22541026

[20] Cañal-Bruland R, Balch L, Niesert L. Judgement bias in predicting the success of one’s own basketball free throws but not those of others. Psychol Res. 2015;79: 548–555. 10.1007/s00426-014-0592-2 24965214

[21] Knoblich G, Flach R. Predicting the Effects of Actions: Interactions of Perception and Action. Psychol Sci. 2001;12: 467–472. 10.1111/1467-9280.00387 11760133

[22] Rieser JJ, Erdemir A, Khuu NT, Beck S. Knowing the Results of One’s Own Actions Without Visual or Auditory Feedback When Walking, Throwing, and Singing. Ecol Psychol. 2014;26: 134–146. 10.1080/10407413.2014.875318

[23] Yazmir B, Reiner M. I act, therefore I err: EEG correlates of success and failure in a virtual throwing game. Int J Psychophysiol. Elsevier B.V; 2015; 10.1016/j.ijpsycho.2017.02.007 28193497

[24] Beets IAM, Macé M, Meesen RLJ, Cuypers K, Levin O, Swinnen SP. Active versus passive training of a complex bimanual task: Is prescriptive proprioceptive information sufficient for inducing motor learning? PLoS One. 2012;7 10.1371/journal.pone.0037687 22666379 PMC3359339

[25] Fuentes CT, Bastian AJ. Where Is Your Arm? Variations in Proprioception Across Space and Tasks. J Neurophysiol. 2010;103: 164–171. 10.1152/jn.00494.2009 19864441 PMC4116392

[26] Sevrez V, Bourdin C. On the Role of Proprioception in Making Free Throws in Basketball. Res Q Exerc Sport. 2015;86: 274–280. 10.1080/02701367.2015.1012578 25775217

[27] Schmidt RA, Lange C, Young DE. Optimizing summary knowledge of results for skill learning. Hum Mov Sci. 1990;9: 325–348. 10.1016/0167-9457(90)90007-Z

[28] Müller S, Abernethy B. Batting with occluded vision: An in situ examination of the information pick-up and interceptive skills of high- and low-skilled cricket batsmen. J Sci Med Sport. 2006;9: 446–458. 10.1016/j.jsams.2006.03.029 16713351

[29] Henderson SE. Predicting the accuracy of a throw without visual feedback. J Hum Mov Stud. United Kingdom: Teviot Scientific Publications; 1975;1: 183–189.

[30] Vickers JN. Visual control when aiming at a far target. J Exp Psychol Hum Percept Perform. 1996;22: 342–354. 10.1037/0096-1523.22.2.342 8934848

[31] Wallace SA, Hagler RW. Knowledge of Performance and the Learning of a Closed Motor Skill. Res Quarterly Am Alliance Heal Phys Educ Recreat Danc. 1979;50: 265–271. 10.1080/10671315.1979.10615609 472467

[32] Magill RA. The influence of augmented feedback on skill learning depends on characteristics of the skill and the learner. Quest. 1994;46: 314–327. 10.1080/00336297.1994.10484129

[33] Salmoni AW, Schmidt RA, Walter CB. Knowledge of results and motor learning: A review and critical reappraisal. Psychol Bull. 1984;95: 355–386. 10.1037/0033-2909.95.3.355 6399752

[34] Cohen J. Statistical Power Analysis for the Behavioural Science. 2nd ed. Hillsdale NJ: Lawrence Erlbaum Associates; 1988.

[35] Macmillan NA, Creelman CD. Detection Theory: A User’s Guide. 2nd ed. Mahwah NJ: Lawrence Erlbaum Associates; 2005 10.1017/CBO9781107415324.004

[36] Green DM, Swets JA. Signal detection theory and psychophysics. Society. 1966;1: 521 10.1901/jeab.1969.12-475

[37] Cañal-Bruland R, Schmidt M. Response bias in judging deceptive movements. Acta Psychol (Amst). Elsevier B.V.; 2009;130: 235–240. 10.1016/j.actpsy.2008.12.009 19193359

[38] Button C, MacLeod M, Sanders R, Coleman S. Examining movement variability in the basketball free-throw action at different skill levels. Res Q Exerc Sport. 2003;74: 257–269. 10.1080/02701367.2003.10609090 14510290

[39] Giardini F, Coricelli G, Joffily M, Sirigu a. Overconfidence in Predictions as an Effect of Desirability Bias. Adv Decis Mak Under Risk Uncertain. 2008; 163–180.

[40] Johnson DDP, Fowler JH. The evolution of overconfidence. Nature. Nature Publishing Group; 2011;477: 317–320. 10.1038/nature10384 21921915

