# Supplementary material for: Influence of skill level on predicting the success of one's own basketball free throws
Source: PLoS One. 2019 Mar 22;14(3):e0214074. doi: 10.1371/journal.pone.0214074 (PMC6430392; doi:10.1371/journal.pone.0214074)
Supplement: S1 Table — (DOCX) [file pone.0214074.s001.docx]

**S1 Table. Experiment data**

| Subject^1^ | Baseline^2^ | Result: in | Result: out | Prediction: in | Prediction: out | TP^3^ | FN^3^ | FP^3^ | TN^3^ |
| --- | --- | --- | --- | --- | --- | --- | --- | --- | --- |
| 1 | 1 | 8 | 22 | 12 | 18 | 4 | 4 | 8 | 14 |
| 2 | 4 | 1 | 29 | 6 | 24 | 0 | 1 | 6 | 23 |
| 3 | 6 | 9 | 21 | 15 | 15 | 5 | 4 | 10 | 11 |
| 4 | 9 | 16 | 14 | 17 | 13 | 11 | 5 | 6 | 8 |
| 5 | 5 | 11 | 19 | 21 | 9 | 9 | 2 | 12 | 7 |
| 6 | 6 | 15 | 15 | 14 | 16 | 10 | 5 | 4 | 11 |
| 7 | 9 | 11 | 19 | 19 | 11 | 9 | 2 | 10 | 9 |
| 8 | 2 | 9 | 21 | 15 | 15 | 5 | 4 | 10 | 11 |
| 9 | 5 | 12 | 18 | 18 | 12 | 8 | 4 | 10 | 8 |
| 10 | 9 | 15 | 15 | 21 | 9 | 11 | 4 | 10 | 5 |
| 11 | 6 | 0 | 30 | 14 | 16 | 0 | 0 | 14 | 16 |
| 12 | 5 | 8 | 22 | 15 | 15 | 4 | 4 | 11 | 11 |
| 13 | 4 | 0 | 30 | 12 | 18 | 0 | 0 | 12 | 18 |
| 14 | 6 | 11 | 19 | 10 | 20 | 5 | 6 | 5 | 14 |
| 15 | 3 | 6 | 24 | 17 | 13 | 6 | 0 | 11 | 13 |
| 16 | 7 | 18 | 12 | 18 | 12 | 12 | 6 | 6 | 6 |
| 17 | 5 | 12 | 18 | 19 | 11 | 9 | 3 | 10 | 8 |
| 18 | 7 | 8 | 22 | 20 | 10 | 6 | 2 | 14 | 8 |
| 19 | 10 | 6 | 24 | 17 | 13 | 5 | 1 | 12 | 12 |
| 20 | 8 | 16 | 14 | 21 | 9 | 11 | 5 | 10 | 4 |
| 21 | 11 | 25 | 5 | 22 | 8 | 19 | 6 | 3 | 2 |
| 22 | 9 | 12 | 18 | 22 | 8 | 10 | 2 | 12 | 6 |
| 23 | 4 | 10 | 20 | 16 | 14 | 8 | 2 | 8 | 12 |
| 24 | 9 | 14 | 16 | 23 | 7 | 14 | 0 | 9 | 7 |
| 25 | 8 | 14 | 16 | 24 | 6 | 14 | 0 | 10 | 6 |
| 26 | 8 | 11 | 19 | 19 | 11 | 8 | 3 | 11 | 8 |
| 27 | 6 | 15 | 15 | 22 | 8 | 13 | 2 | 9 | 6 |
| 28 | 9 | 16 | 14 | 21 | 9 | 13 | 3 | 8 | 6 |
| 29 | 13 | 12 | 18 | 23 | 7 | 8 | 4 | 15 | 3 |
| 30 | 9 | 14 | 16 | 18 | 12 | 11 | 3 | 7 | 9 |
| 31 | 10 | 20 | 10 | 23 | 7 | 19 | 1 | 4 | 6 |
| 32 | 11 | 22 | 8 | 26 | 4 | 19 | 3 | 7 | 1 |
| 33 | 13 | 20 | 9 | 23 | 6 | 17 | 3 | 6 | 3 |
| 34 | 12 | 23 | 7 | 26 | 4 | 20 | 3 | 6 | 1 |
| 35 | 11 | 5 | 25 | 21 | 9 | 5 | 0 | 16 | 9 |
| 36 | 12 | 19 | 11 | 26 | 4 | 16 | 3 | 10 | 1 |
| 37 | 8 | 16 | 14 | 21 | 9 | 12 | 4 | 9 | 5 |
| 38 | 12 | 5 | 25 | 12 | 18 | 4 | 1 | 8 | 17 |
| 39 | 10 | 11 | 19 | 17 | 13 | 7 | 4 | 10 | 9 |
| 40 | 11 | 18 | 12 | 11 | 19 | 9 | 9 | 2 | 10 |

1. Recreational subjects: 1 - 20; Collegiate subjects: 21 -40
2. Baseline: number of made shots out of 15
3. TP = true positive (result in, prediction in); FN = false negative (result in, prediction out); FP = false positive (result out, prediction in); TN = true negative (result out, prediction out)
